# AI-Supported Semiquantitative CT Characterization of COVID-19 Pneumonia Across SARS-CoV-2 Wild-Type, Alpha, and Delta Variants and Its Association with Clinical Parameters

**DOI:** 10.3390/diagnostics16213696

**Published:** 2026-11-09

**Authors:** Meike S. Schmidberger, Stefan A. Schmidt, Susanne Martina Büttner, Franziska Flack, Bettina Jungwirth, Beate Grüner, Steffen Stenger, Thomas Stamminger, Meinrad Beer

**Affiliations:** 1 Department of Diagnostic and Interventional Radiology, Ulm University Medical Center, Albert-Einstein-Allee 23, 89081 Ulm, Germany; 2 Scientific Collaborations Siemens Healthineers AG, Siemensstraße 3, 91301 Forchheim, Germany; 3 Department of Anesthesiology and Intensive Care Medicine, Ulm University Medical Center, Albert-Einstein-Allee 23, 89081 Ulm, Germany; 4 Division of Infectious Diseases, Department of Internal Medicine III, Ulm University Medical Center, 89081 Ulm, Germany; 5 Institute for Medical Microbiology and Hygiene, Ulm University Medical Center, 89081 Ulm, Germany; 6 Institute for Virology, Ulm University Medical Center, 89081 Ulm, Germany

**Keywords:** COVID-19 pneumonia, computed tomography, artificial intelligence, quantitative imaging, pulmonary opacity, SARS-CoV-2 variants

## Abstract

**Objective:** An AI-based software prototype for the detection of coronavirus disease 2019 (COVID-19) pneumonia was evaluated regarding its potential to quantify pulmonary opacities across Severe Acute Respiratory Syndrome Coronavirus 2 (SARS-CoV-2) variant groups, incorporating clinical and radiological parameters to facilitate faster diagnosis and therapy. **Methods:** Chest computed tomography (CT) examinations of 255 patients with confirmed COVID-19 pneumonia were analyzed using an AI-supported semiquantitative CT software prototype between November 2020 and December 2021. The results were correlated with clinical and laboratory parameters. The cohort consisted of wild-type (*n* = 93), Alpha (*n* = 66), Beta/Gamma (*n* = 3), and Delta (*n* = 93). Beta/Gamma cases were excluded from subgroup comparisons due to the small number of patients. **Results:** The mean AI-derived COVID-19 probability was 0.84 ± 0.33. After exclusion of five technically non-evaluable measurements, global mean opacity attenuation differed significantly among the wild-type (−475.12 ± 116.47 Hounsfield Units (HU)), Alpha (−464.68 ± 114.80 HU), and Delta groups (−423.92 ± 112.56 HU; Kruskal–Wallis H(2) = 12.062, *p* = 0.002). The Volume of Opacity showed no significant differences between wild-type (984.64 ± 908.92 mL), Alpha (1161.17 ± 933.55 mL), and Delta (988.19 ± 866.20 mL) (*p* = 0.339). The extent of pulmonary involvement (Volume of Opacity) correlated with the duration of hospital stay (r = 0.311, *p* < 0.001**), duration of intensive care treatment (r = 0.468, *p* < 0.001**), and the laboratory parameters CRP, leukocyte count, relative lymphocyte count, and AST (*p* < 0.001**). It showed no significant correlation with the duration of invasive ventilation (*p* > 0.05). A total of 37 (14.5%) patients died and 92 (36.1%) required intensive care unit (ICU) admission. Manual corrections of lung lobe and infiltrate boundaries were performed in 221/255 datasets and were required in all assessed datasets (221/221, 100%). **Conclusions:** The CT Pneumonia Analysis Prototype enabled semiquantitative assessment of pulmonary opacities across different SARS-CoV-2 variants but required radiological oversight and manual correction of the automated segmentations. Given the retrospective observational design, the findings should be interpreted in light of potential confounding and heterogeneity in CT acquisition protocols. The observed associations suggest that combining semiquantitative CT parameters with clinical and laboratory data may be of potential relevance for future prognostic models, which requires further evaluation in multivariable analyses.

## 1. Introduction

In December 2019, the beta-coronavirus SARS-CoV-2 (Severe Acute Respiratory Syndrome Coronavirus 2) was discovered in Wuhan, China. The resulting disease, COVID-19 (coronavirus disease 2019) was characterized as a pandemic by the WHO (World Health Organization) on March 11, 2020, following its global spread [1]. COVID-19 led to more than 775 million reported cases and over 7 million reported deaths worldwide [2,3].

In 2021, lower respiratory tract infections ranked fifth among global causes of death and, alongside COVID-19, were among the deadliest communicable diseases worldwide [4].

In Germany, COVID-19 was also among the leading causes of death in 2021 and the leading infectious disease in both pandemic years [5,6]. The pandemic progressed in multiple waves, with the original SARS-CoV-2 wild-type (WT) dominating in 2020, followed by the emergence of successive genetic variants from 2021 onward [7].

In the early phase of the pandemic, radiological images were used to diagnose COVID-19 due to limited PCR test resources [8,9,10,11]. While polymerase chain reaction (PCR) remains the gold standard for pathogen detection due to its high sensitivity and specificity [7], rapid antigen tests provided an additional but less sensitive diagnostic option. Nevertheless, computed tomography (CT) imaging has retained considerable clinical relevance, both in severe COVID-19 cases and in the diagnostic workup of Long COVID [12,13].

Radiological imaging plays a central role in diagnosis worldwide [14,15]. However, its availability varies substantially by region, while resources for microbiological testing remain limited in many settings [14,16]. Meanwhile, the growing global shortage of radiology workforce, including radiographers and radiologists, poses significant challenges to timely and high-quality radiological assessment [17,18], underscoring the urgency of advancing artificial intelligence (AI) in radiological diagnostics.

Conventional radiological reporting is performed by radiologists and is inherently subject to interindividual variability and subjectivity. Computer-aided detection (CAD) systems, often powered by AI-based convolutional neural networks (CNNs), enable automated quantitative image analysis [19]. A distinction is drawn between fully automated AI-driven image evaluation and semi-automated approaches, which combine algorithmic systems with conventional radiological interpretation [19]. Such AI-based methods are typically disease- and modality-specific and have been applied to pneumonia detection and classification [19,20]. In the context of COVID-19 pneumonia, AI-assisted systems have demonstrated the ability to identify characteristic imaging patterns with high sensitivity and specificity, and to differentiate COVID-19 from other pneumonic entities [21,22,23]. Their application is intended to reduce diagnostic errors and radiologists’ workload, and facilitate faster, more standardized, and objective analysis of increasingly large imaging datasets.

The aim of this retrospective single-center study was to evaluate, using an AI-based software prototype for COVID-19 detection, whether patient groups defined by SARS-CoV-2 variant differ in the extent and mean CT attenuation of pulmonary opacities, and how these differences are reflected in AI-derived imaging parameters. Additionally, we examined whether clinical and laboratory parameters differ among SARS-CoV-2 variants, how they are associated with AI-derived imaging parameters, and whether these associations vary by variant.

## 2. Materials and Methods

### 2.1. Ethical Considerations

This retrospective study was approved by the Ethics Committee of Ulm University under reference number 320/21. Given the retrospective study design, written informed consent was waived.

### 2.2. Study Population

**Figure 1:**
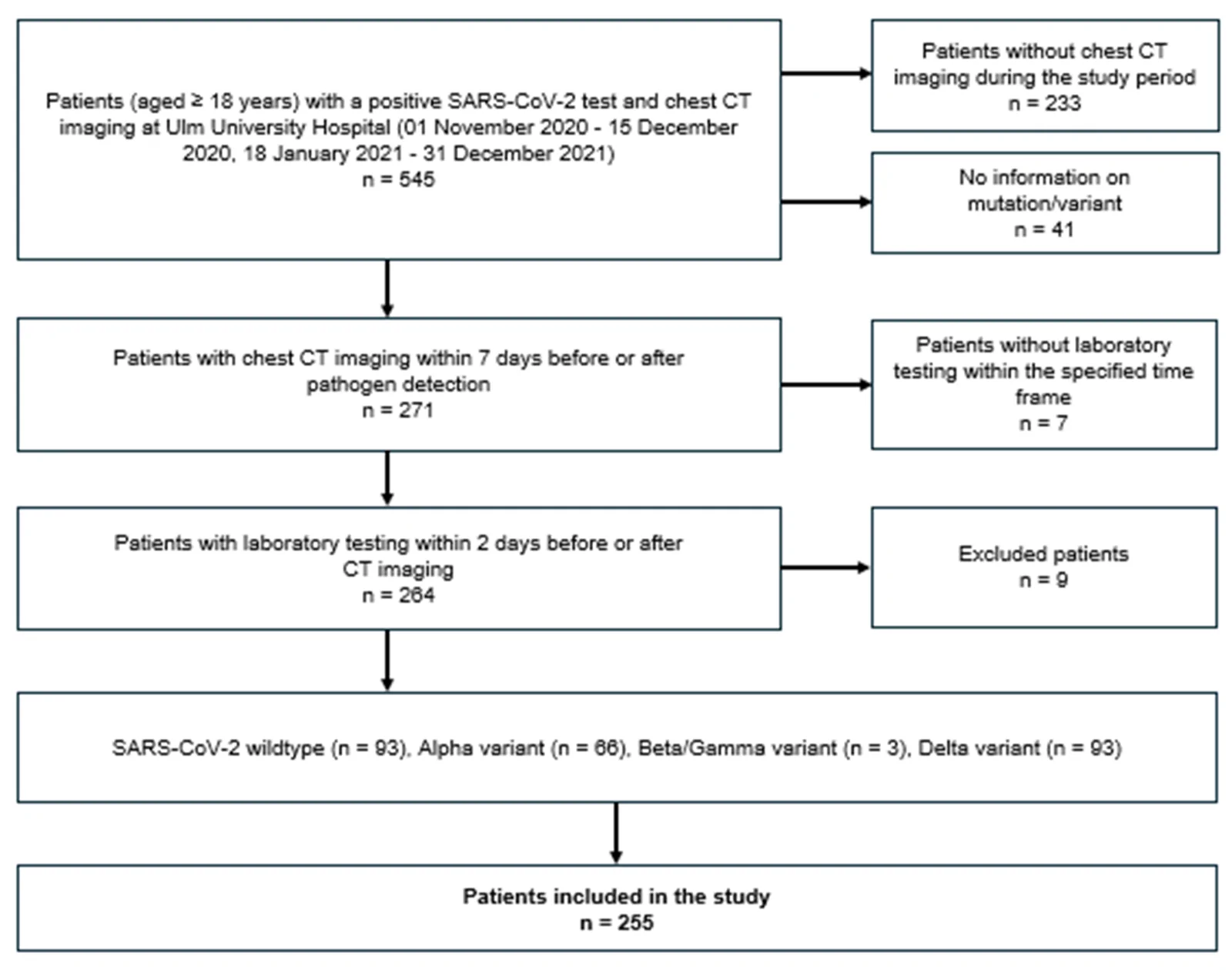
Patient recruitment.

This single-center retrospective study analyzed 255 patients with pulmonary CT datasets and a confirmed SARS-CoV-2 diagnosis, collected between November 2020 and December 2021, excluding the period from 16 December 2020 to 17 January 2021, as the distinction between wild-type and variant infections was unclear. The study comprised the overall cohort as well as subgroups defined by SARS-CoV-2 wild-type/variant determined by whole-genome sequencing or genotypic sequence analysis: Wild-type, *n* = 93; Alpha variant, *n* = 66; Beta/Gamma variants, *n* = 3; Delta variant, *n* = 93. Patient recruitment is illustrated in Figure 1. Exclusion criteria were the absence of a chest CT examination within 7 days before or after a positive SARS-CoV-2 test, the absence of laboratory measurements within ±2 days of CT acquisition, age below 18 years, CT slice thickness > 5 mm, or manual correction time > 20 min (*n* = 3).

### 2.3. CT Examinations

CT image acquisition was performed using seven different scanner models: Siemens EDGE Plus (*n* = 211), Siemens AS+ (*n* = 10), Siemens Force (*n* = 23), Siemens go.TOP (*n* = 1), Siemens Edge (*n* = 1), Toshiba Aquilion (*n* = 7), and Siemens Biograph 40 (*n* = 2). 10 examinations, included in the study were external referrals from other institutions. Slice thickness ranged from 0.75 to 5 mm, with 96.4% being ≤ 1.0 mm. The mean tube voltage was 110.12 ± 15.9 kV, and the mean tube current-time product was 77.52 ± 56.63 mAs. Complete pulmonary imaging was achieved in 246 patients (96.5%). Contrast agent was administered to 94 patients (36.9%). Among the 252 patients included in the three main variant groups, contrast-enhanced CT was performed in 24/93 wild-type patients (25.8%), 30/66 Alpha patients (45.5%), and 38/93 Delta patients (40.9%). Detailed CT acquisition characteristics stratified by SARS-CoV-2 variant are provided in supplementary Table S1. Image processing using the CT Pneumonia Analysis Prototype was performed in the axial plane using lung window settings.

### 2.4. Image Analysis

**Figure 2:**
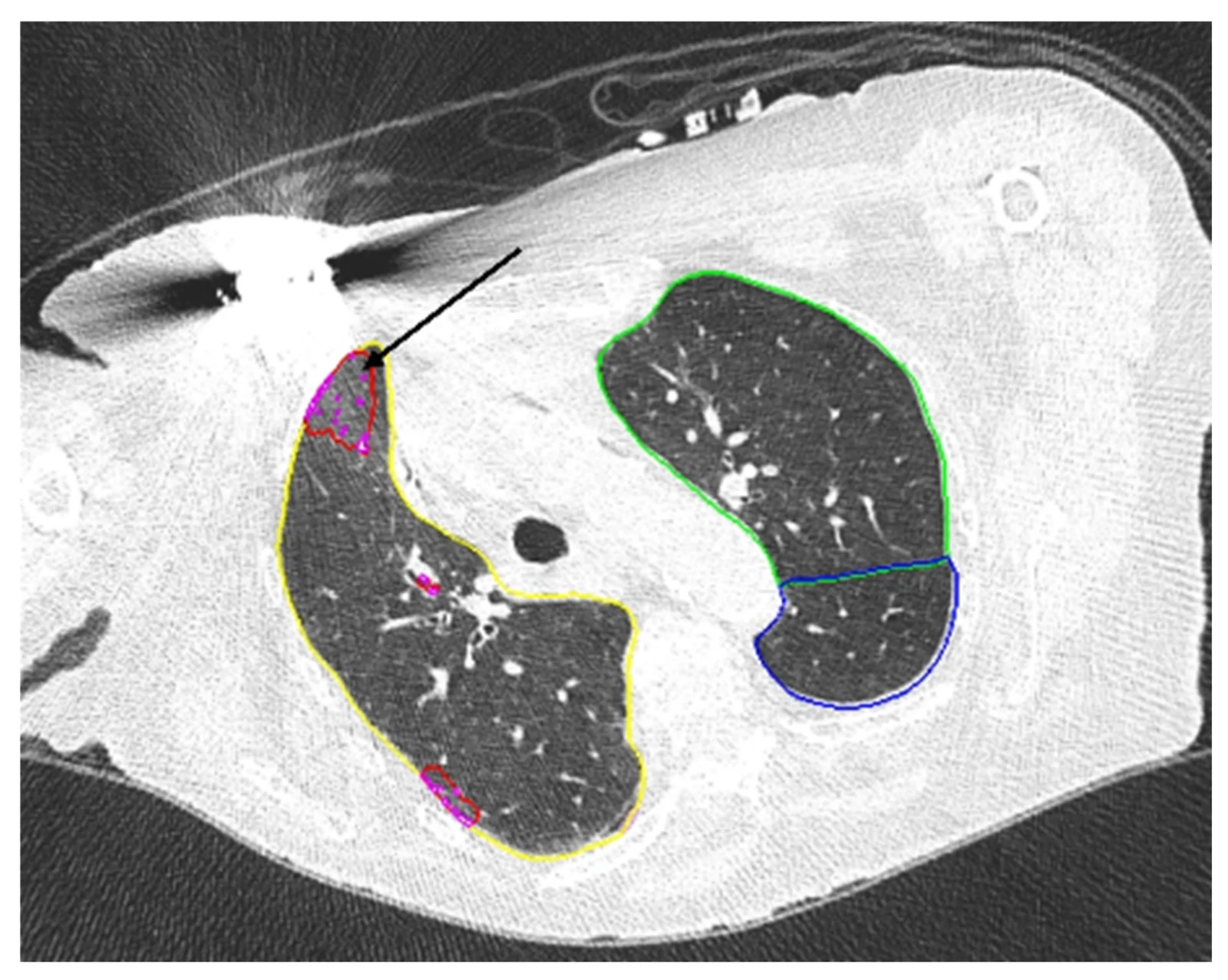
Axial computed tomography image in the lung window setting. The segmentation of a radiation artifact (outlined in red; see arrow) in the left upper lobe (outlined in yellow) caused by a radiopaque foreign body is shown. Source: own figure, Department of Diagnostic and Interventional Radiology, Ulm University Hospital.

**Figure 3:**
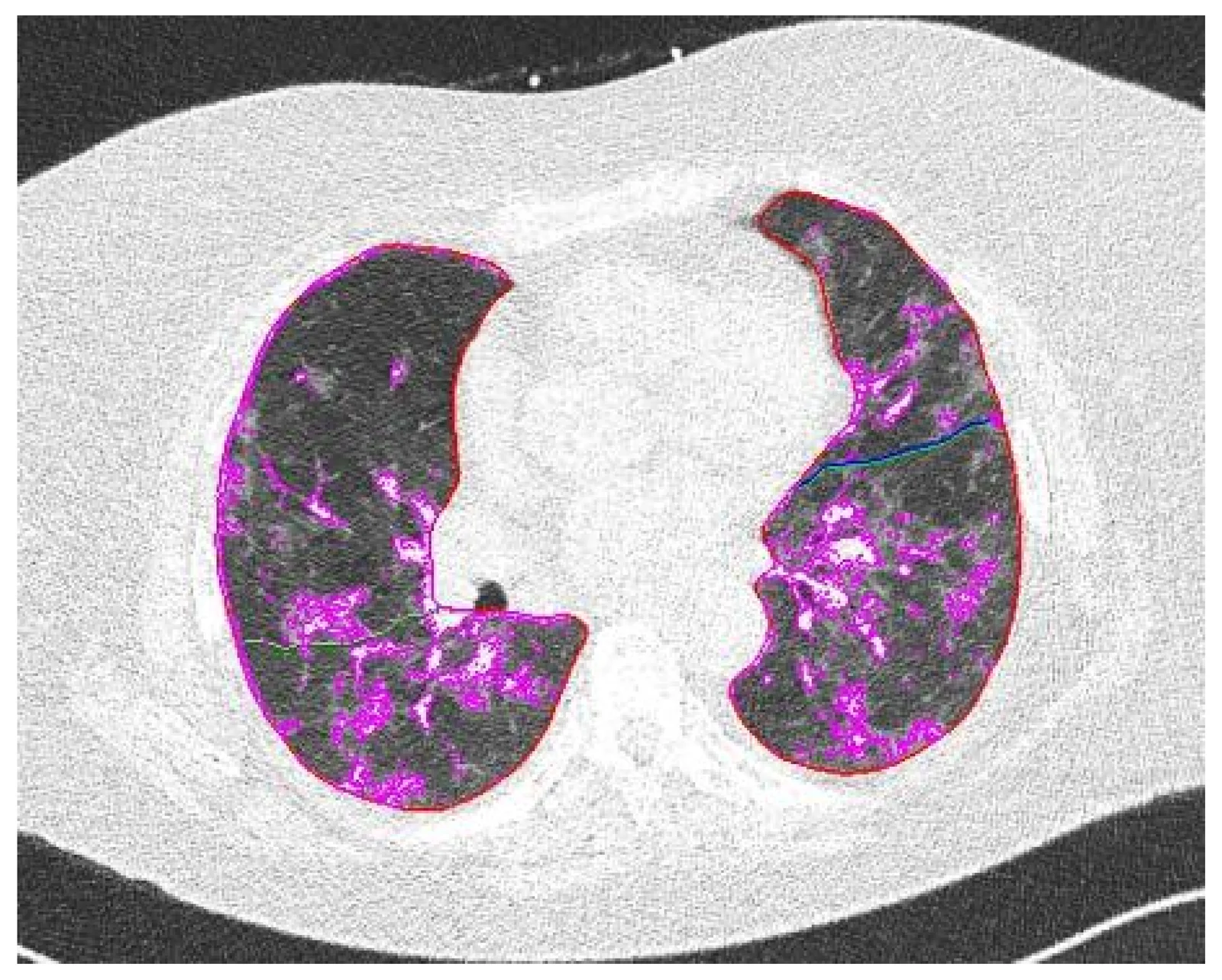
Axial computed tomography image in the lung window setting showing excessive segmentation (outlined in red) involving unaffected lung parenchyma (false positives). Source: own figure, Department of Diagnostic and Interventional Radiology, Ulm University Hospital.

CT image data were processed using the syngo.via CT Pneumonia Analysis Prototype (Version 1.0.4.2, Siemens Healthineers, Erlangen, Germany), a deep learning algorithm intended exclusively for research purposes and not for clinical use. The prototype identifies and quantifies pulmonary regions of abnormally increased attenuation [24]. Algorithm development and validation were based on 9749 non-contrast chest CT volumes. Pulmonary lesion segmentation is based on Dense U-Net, a specialized convolutional neural network (CNN) that detects, analyzes, and three-dimensionally segments ground-glass opacities and consolidations [25]. CT image data were imported from the DeepUnity Diagnost (version 1.1.0.1 and 1.1.1.1, Dedalus, Bonn, Germany) imaging software in DICOM (Digital Imaging and Communications in Medicine) format into the syngo.via platform and accessed via the MM Research Frontier Server using the CT Pneumonia Analysis Prototype application. Following automated AI analysis, lung lobe segmentations and pulmonary infiltrates, could be manually corrected to remove artifacts, pleural effusions, contrast-enhanced vessels, and false-positive annotations (Figure 2 and Figure 3).

Image analysis was performed under single-blinded conditions, with the datasets evaluated in randomized order. Following the initial assessment by the first reader (M.S.S.), all analyses were reviewed and, if necessary, corrected by a second reader (M.B.) with more than 25 years of professional experience in thoracic radiology. No comparative analysis between the first and second reader was performed. Manual corrections were classified as “major” (≥2 min) or “minor” (<2 min). The extent of manual correction was assessed in 221 of the 255 datasets.

**Figure 4:**
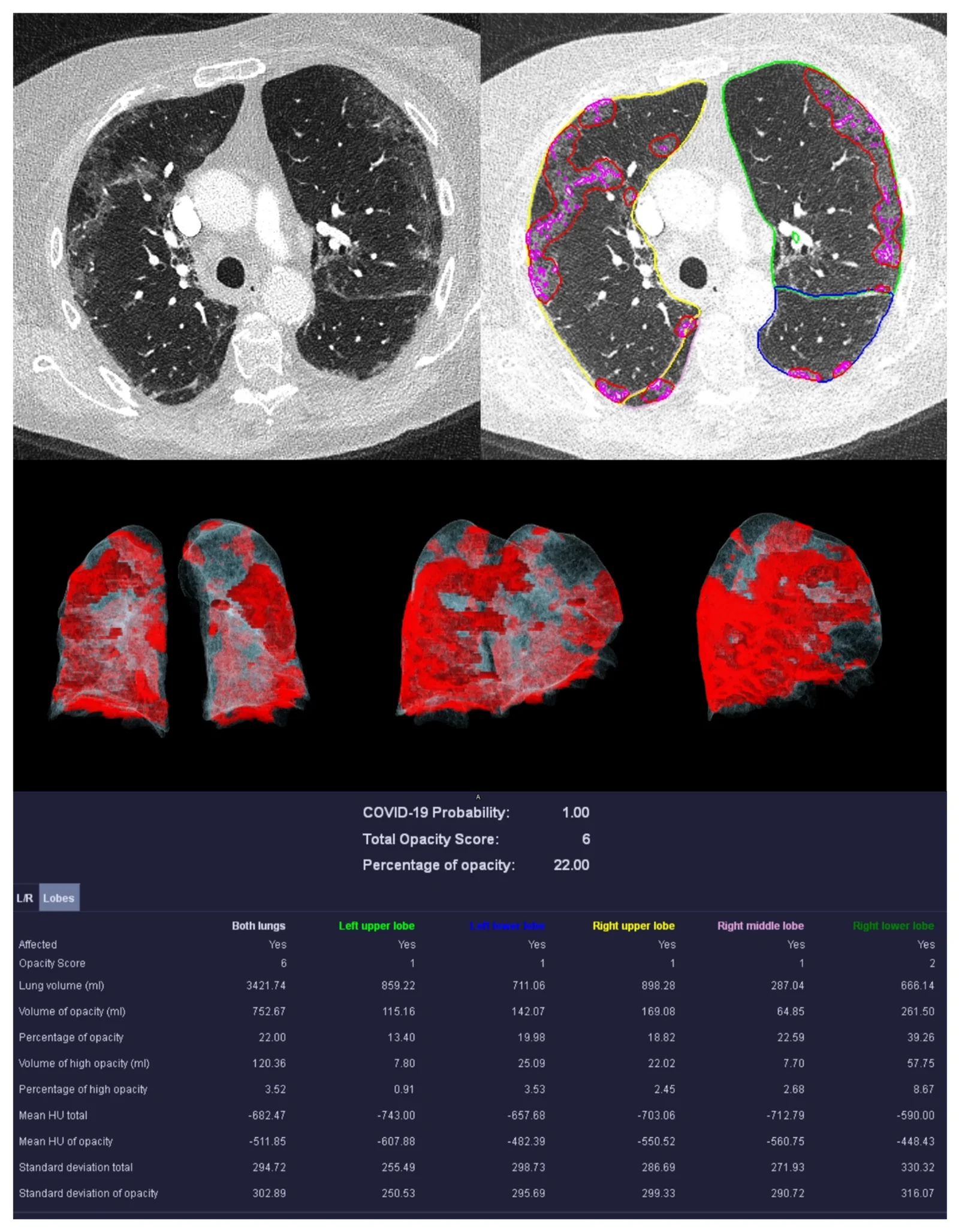
Results of CT image analysis using the CT Pneumonia Analysis Prototype in an 83−year-old patient with Alpha variant COVID-19 pneumonia (2020). Bilateral peripheral, ill−defined pulmonary infiltrates (red) are shown, along with native and segmented axial lung images, three−dimensional reconstructions, and lobe-based semiquantitative analysis. Source: own figure, Department of Diagnostic and Interventional Radiology, Ulm University Hospital.

Upon completion, the AI generated multiplanar lung visualizations and tabular summaries of semiquantitative parameters (Figure 4). The COVID-19 Probability score ranges from 0 to 1 and reflects the likelihood of COVID-19 pneumonia. Volumes of the lung, lung lobes, and infiltrates are reported in milliliters and percentages. Mean infiltrate density is expressed in HU [26]. Regions with attenuation ≥ −200 HU (Hounsfield units) are defined as high-density pulmonary areas [24]. Total processing time was recorded for workflow analysis.

### 2.5. Radiological Image Quality Assessment

The study investigator and an independent reviewer assessed all 255 CT datasets using the PGMI image-quality classification perfect, good, moderate, and inadequate, considering motion and radiation artifacts, and the completeness of pulmonary coverage.

### 2.6. Virological Reference Standard

All study participants were tested positive for SARS-CoV-2 by reverse transcription polymerase chain reaction (RT-PCR) at Ulm University Hospital. SARS-CoV-2 variant assignment was based on whole-genome sequencing or genotypic sequence analysis.

### 2.7. Study Design

Clinical and laboratory data were retrieved from the institutional SAP system. Clinical variables included length of hospital stay, length of intensive care unit (ICU) stay, duration of invasive mechanical ventilation, and discharge status (deceased, recovered, or transferred). Laboratory values obtained within ±2 days of CT examination were used: CK (creatine kinase), Troponin T, LDH (lactate dehydrogenase), AST (aspartate aminotransferase), ALT (alanine aminotransferase), CRP (C-reactive protein), leukocytes, lymphocytes, D-dimer, procalcitonin, IL-6 (interleukin-6), lactate, and venous blood gas values including venous pH, venous pCO_2_ (partial pressure of carbon dioxide), venous pO_2_ (partial pressure of oxygen), and venous oxygen saturation. These parameters were analyzed for associations with automated segmentation findings and examined across viral subgroups. Comorbidities were categorized as cardiometabolic (cardiovascular disease, diabetes mellitus, arterial hypertension and hyperlipoproteinemia) or chronic organ diseases (chronic obstructive pulmonary disease, COPD), other chronic pulmonary conditions, and chronic renal disease) and assessed for significant differences between viral subtypes.

### 2.8. Statistical Analysis

Data processing and statistical analysis were performed using Excel 2024 (Microsoft, Redmond, USA) and IBM SPSS Statistics, versions 30 and 31 (IBM Corp., Armonk, NY, USA). Qualitative variables are presented as absolute and relative frequencies; metric variables as mean ± standard deviation (SD). Normal distribution was assessed using the Shapiro–Wilk test. Significance thresholds were set at 5% and 1%. Non-normally distributed variables were analyzed using appropriate non-parametric tests and correlations were calculated using Spearman’s rank correlation coefficient (Spearman’s rho). Spearman’s correlation coefficients were used as measures of association strength. Given the exploratory nature and the number of correlation analyses, the corresponding *p*-values were not adjusted for multiple testing and should be interpreted descriptively rather than as confirmatory evidence. Group differences across viral subtypes for ordinal and metric parameters were assessed using the Kruskal–Wallis test, with pairwise comparisons performed using two-sided Mann–Whitney U tests. For the three pairwise comparisons of global mean opacity attenuation, statistical significance was assessed after Bonferroni correction. The *p* values reported for global and regional pairwise comparisons are unadjusted and should be interpreted as exploratory. As a sensitivity analysis addressing potential confounding by contrast administration, comparisons of global mean opacity attenuation were repeated in the subgroup of patients who underwent non-contrast-enhanced CT. Five technically non-evaluable global attenuation measurements were treated as missing in the corresponding analyses, without excluding these patients from analyses of other parameters.

For the primary global opacity attenuation analysis, rank-based effect sizes were calculated using epsilon-squared (ε^2^) for Kruskal–Wallis tests and r for Mann–Whitney U tests. Unadjusted 95% confidence intervals for differences in group means were estimated using Welch’s method.

## 3. Results

### 3.1. Patient Cohort

A total of 255 patients from the Ulm University Hospital (146 males and 109 females; mean age 63.6 ± 15.6 years, range 23–97 years) with lung CT datasets acquired between November 2020 and December 2021 (excluding 16 December 2020 to17 January 2021) were analyzed. The mean interval between virological confirmation of SARS-CoV-2 infection and CT imaging was −0.09 ± 1.74 days; negative values indicate that CT was performed prior to virological confirmation. The Beta/Gamma variant was excluded from subgroup comparisons due to the small sample size (*n* = 3).

In total, 88.6% (*n* = 226) of patients had comorbidities in addition to COVID-19. Cardiometabolic diseases were present in 72.2% (*n* = 184), chronic organ diseases in 26.7% (*n* = 68), and immunosuppression in 12.5% (*n* = 32).

### 3.2. Clinical Course

The mean duration of hospitalization was 12.47 ± 9.54 days. Overall, 92 patients (36.1%) required admission to the intensive care unit (ICU) with a mean ICU stay of 12.17 ± 9.91 days (range 1–52 days). Supplemental oxygen administered via nasal cannula or mask was required in 187 patients (73.3%), whereas 84 patients (32.9%) received non-invasive ventilation (NIV) or high-flow oxygen therapy (HFOT). Invasive mechanical ventilation via endotracheal intubation or tracheostomy) was required in 37 patients (14.5%) for a mean duration of 14.97 ± 10.19 days (range, 1–43 days). Among the 255 patients, 37 (14.5%) died, 181 (71.0%) recovered, and 36 (14.1%) were transferred to other hospitals, outcome data were unavailable for one patient (0.4%). None of these clinical parameters differed significantly among the viral subgroups (all *p* > 0.05). Detailed subgroup distributions are provided in Table 1.

### 3.3. Laboratory Parameters

An overview of laboratory parameters and subgroup stratification is shown in Table 2. AST levels were significantly lower in the wild-type compared to Alpha (*p* = 0.004**) and Delta (*p* = 0.040*). In addition D-dimer levels were significantly lower in the wild-type compared to Alpha (*p* = 0.001**) and Delta (*p* = 0.001**), although mean values in all groups exceeded the normal range. Venous blood gas pH differed significantly between subgroups (*p* = 0.026*). No significant differences were observed for other laboratory parameters (*p* > 0.05).

### 3.4. Image Analysis

Among the 221 datasets assessed for correction extent, major manual corrections were required in 64 (29%), whereas minor corrections were required in 157 (71%). Irrespective of the extent of manual correction, complete resegmentation was necessary in 24/255 datasets (9.4%). Image quality did not differ significantly between subgroups of virus variants (*p* = 0.345). Mean processing times were 0.54 ± 2.07 min for image import, 2.36 ± 0.63 min for automated segmentation, 1.96 ± 2.53 min for post-processing, and 1.56 ± 0.56 min for saving/export, yielding a mean total processing time of 6.42 ± 3.92 min. Mean post-processing time was significantly longer for the Alpha variant (2.59 ± 2.84 min) than for the wild-type (2.21 ± 3.36 min; *p* = 0.011*) and Delta variant (1.41 ± 1.21 min; *p* < 0.001**). Similarly, mean total processing time was significantly longer for the Alpha variant (7.42 ± 3.38 min) than for the wild-type (6.71 ± 5.98 min; *p* = 0.001**) and Delta variant (5.59 ± 1.35 min; *p* < 0.001**).

### 3.5. Semiquantitative AI-Supported CT Analysis

The AI-derived COVID-19 probability score showed a markedly left-skewed distribution, with a mean of 0.84 ± 0.33, a median of 1.00 (interquartile range: 0.96–1.00), and a range of 0.00–1.00. A score of 1.00 was recorded in 153 of 255 patients (60.0%), indicating a pronounced ceiling effect. No statistically significant differences were observed between the variant groups (*p* = 0.059). Total lung volume was 3777.02 ± 1160.62 mL, and total opacity volume was 1033.61 ± 899.98 mL. Analyses of COVID-19 probability, opacity density (HU), and opacity volume (mL and %) across SARS-CoV-2 subgroups are presented in Table 3 (global and lobar analysis).

No subgroup differences were observed for volume (ml; *p* = 0.339) or percentage of lung involvement (*p* = 0.257). Following exclusion of five technically non-evaluable global HU measurements, global mean opacity attenuation differed significantly among the wild-type, Alpha, and Delta groups (−475.12 ± 116.47 HU, *n* = 92; −464.68 ± 114.80 HU, *n* = 66; and −423.92 ± 112.56 HU, *n* = 89, respectively; Kruskal–Wallis H(2) = 12.062, *p* = 0.002). The rank-based effect size was small (ε^2^ = 0.041). The mean attenuation difference between Delta and wild-type was 51.20 HU (unadjusted 95% CI: 17.60–84.79 HU).

In a sensitivity analysis restricted to non-contrast-enhanced CT examinations (*n* = 157), mean opacity attenuation was −504.83 ± 99.50 HU in the wild-type group (*n* = 68), −458.59 ± 110.26 HU in the Alpha group (*n* = 36), and −427.85 ± 120.89 HU in the Delta group (*n* = 53). Differences across groups remained significant (Kruskal–Wallis H(2) = 16.510, *p* < 0.001), including the comparison between wild-type and Delta (Mann–Whitney U = 1027, *p* < 0.001). In this non-contrast-enhanced subgroup, the rank-based effect size was ε^2^ = 0.094 for the overall comparison and r = 0.368 for the wild-type versus Delta comparison. The mean attenuation difference between Delta and wild-type was 76.98 HU (unadjusted 95% CI: 36.26–117.70 HU).

Table 4 presents unadjusted *p*-values for exploratory pairwise comparisons of mean opacity attenuation across lung regions. Among the three global pairwise comparisons, only the difference between wild-type and Delta remained statistically significant after Bonferroni correction.

### 3.6. Correlation of Clinical and Laboratory Parameters with Semiquantitative Imaging Parameters

In the overall cohort, opacity volume showed highly significant weak to moderate correlations with length of hospital stay (r = 0.311; *p* < 0.001**) and ICU stay (r = 0.468; *p* < 0.001**), but not with duration of invasive ventilation. Subgroup analyses (Table 5) revealed highly significant correlations (*p* < 0.01) for wild-type and significant correlations (*p* < 0.05) for Alpha and Delta; for Delta, only hospital length of stay correlated.

Opacity volume showed predominantly highly significant correlations with CRP (r = 0.626; *p* < 0.001**), leukocyte count (r = 0.332; *p* < 0.001**), relative lymphocyte count (r = −0.348; *p* < 0.001**), and AST (r = 0.518; *p* < 0.001**). No significant correlations were found for D-dimer levels (*p* > 0.05). Similar patterns of predominantly highly significant weak to strong correlations were observed within subgroups (see Table 6).

## 4. Discussion

### 4.1. Introduction

During the COVID-19 pandemic, thoracic CT played a crucial diagnostic role, particularly during the early phase when testing capacity was limited. In our retrospective study, an AI-assisted prototype trained on CT examinations of COVID-19 pneumonia provided semiquantitative analysis of pulmonary involvement across SARS-CoV-2 subgroups and identified associations with clinical and laboratory parameters. Furthermore, quantitative CT parameters were compared across variant groups, and the distribution of the AI-derived COVID-19 probability score was characterized. Importantly, the automated segmentations required radiological review and manual correction, emphasizing the semi-automated rather than fully automated nature of the workflow. Nevertheless, our findings highlight the potential of such radiologist-supervised, AI-assisted quantification of COVID-19-associated pulmonary opacities on CT, particularly when considered in conjunction with clinical and laboratory parameters.

### 4.2. Study Population

The high prevalence of comorbidities in our cohort (88.6%) is consistent with the S3 guideline for inpatient COVID-19 treatment, which likewise reports high rates of pre-existing cardiovascular, metabolic, and pulmonary conditions among hospitalized patients [27]. By contrast, Hu et al. reported comorbidities in only 45.8% of patients and found no differences between the wild-type and Delta groups [28]. Similarly, no subgroup-specific differences were observed in the present study. The higher prevalence in our cohort may reflect differences in the definitions used to classify comorbidities.

### 4.3. Clinical Parameters

A study based on data from 14 German university hospitals (09/2020) reported a mortality rate of 18.82% and a mean hospital stay of 18.10 ± 18.75 days [29]. Both measures were lower in our cohort, at 14.5% and 12.47 ± 9.54 days, respectively. These differences may reflect site-specific factors, changes in treatment, and vaccination-related effects associated with the later study period. Fisman et al. and Ong et al. reported more severe disease associated with the Delta variant [30,31]. In contrast, the present cohort showed no significant subgroup differences, potentially because of limited statistical power in some subgroups and unmeasured confounding factors, including vaccination status [27].

### 4.4. Laboratory Parameters

Significant differences among the wild-type, Alpha, and Delta groups were observed for D-dimer, AST, and venous pH. D-dimer and AST levels were lower in the wild-type group, whereas the remaining laboratory parameters did not differ significantly. Similar differences have been reported elsewhere, although they were no longer significant after adjustment for covariates [32]. These findings may therefore reflect differences in inflammatory activity, comorbidities, disease severity, or hepatic involvement rather than variant-specific effects of COVID-19.

### 4.5. Semiquantitative AI-Generated Analysis

Semiquantitative CT analysis demonstrated higher (less negative) global mean opacity attenuation in the Delta group compared with the wild-type group. This difference persisted in the sensitivity analysis restricted to non-contrast-enhanced CT examinations and remained statistically significant after correction for the three global pairwise comparisons. In contrast, neither global comparison involving Alpha remained significant after Bonferroni correction.

These findings describe differences in the CT attenuation of segmented pulmonary opacities but do not demonstrate greater clinical disease severity, faster disease progression, or an independent causal effect of the viral variant. Inui et al.

Inui et al. previously described denser pulmonary infiltrates with increased consolidations and reticular changes in patients infected with the Delta variant [33]. Potential biological mechanisms, including enhanced cell fusion and increased pathogenicity, have also been discussed in the literature [34]. However, given the close temporal relationship between SARS-CoV-2 variants and the respective pandemic phases, time-dependent confounding cannot be excluded, and the present retrospective data do not permit the observed attenuation differences to be attributed exclusively to variant-specific biology or conclusions to be drawn regarding the underlying mechanisms.

Variant-independent studies report that lower lobes, particularly the right, are frequently affected by typical COVID-19 lesions [35,36]. This can be explained by gravity-dependent accumulation of fluid and inflammatory cells in the largest lung lobe [37]. Comparable accumulation across all subgroups may overlay potential variant-specific density differences. Opacity volume did not differentiate between subgroups but has been associated with disease course in previous studies [24,38,39]. AI-assisted semi-automated analysis offers rapid quantification, enabling semiquantitative characterization of subtle density differences between variants, while requiring radiological review and manual correction to ensure accurate segmentation. With further development and validation, such approaches may potentially contribute to future risk stratification when integrated with clinical and laboratory parameters.

The AI-derived COVID-19 probability score did not differ significantly among the wild-type, Alpha, and Delta groups (*p* = 0.059). As all included patients had confirmed COVID-19, diagnostic accuracy and equivalence of detection performance across variant groups could not be established. The mean COVID-19 probability was 0.84 ± 0.33, slightly higher than that reported by Ebong et al., who used the same software and observed significant differences between COVID-19 pneumonia, other pneumonias, and healthy controls [21].

### 4.6. Correlation of Clinical and Laboratory Parameters with Semiquantitative AI Parameters

Correlations between AI-supported quantification parameters and clinical and laboratory variables indicate statistical associations but do not establish independent prognostic value. Previous studies have reported associations between the volume and proportion of pulmonary opacities and disease course [39]. The strength and frequency of correlations between lung infiltrate extent and clinical parameters such as hospital and ICU length of stay varied markedly by subgroup. The stronger correlations observed in the wild-type group should be interpreted cautiously, particularly given the differences between pandemic periods and the absence of multivariable adjustment. Among the laboratory parameters, CRP and AST correlated with opacity volume and may reflect the extent of pulmonary involvement, whereas D-dimer showed no significant correlation with opacity volume in this cohort. Overall, these findings suggest that AI-supported quantitative CT parameters, particularly when considered together with clinical and laboratory parameters, may have potential relevance as components of future prognostic models, although their independent prognostic value was not evaluated in the present study.

### 4.7. Image Analysis, Image Quality, and Post-Processing

With regard to technical performance, manual post-processing was required following automated segmentation in 100% of datasets, with a mean duration of 1.96 min. Similarly, Ebong et al. reported routine post-processing with a mean correction time of 3.4 min [21], whereas Chaganti et al. reported approximately 10 s of algorithm editing without specifying whether manual intervention was required [25]. The consistently high manual post-processing requirement in the present study underscores the continued need for human oversight and supports the use of a semi-automated approach. Post-processing and total editing times were significantly longer for the Alpha variant than for the wild-type and Delta variants, potentially reflecting more complex radiological manifestations or greater challenges for the algorithm. Image quality scores did not differ significantly among subgroups, indicating an absence of variant-specific effects on image quality.

### 4.8. Potential and Limitations

Previous studies have reported prognostic associations between quantitative CT parameters, such as the volume and distribution of lung opacities, and disease course [24,38,39].

AI-based systems have demonstrated high sensitivity and specificity [21,22,23] and enable rapid, reproducible, and objective quantification of pulmonary involvement, as well as the detection of subtle imaging patterns [25,40,41].

Multimodal approaches integrating clinical and laboratory variables may further improve predictive performance, as demonstrated by Lassau et al. [41]. The observed correlations between AI-derived CT parameters and clinical and laboratory parameters indicate potentially relevant associations; however, they do not establish independent prognostic value. Nevertheless, manual post-processing was required for all datasets, indicating that fully automated analysis has not yet been achieved.

Our study has several limitations that must be discussed:

First, the single-center, retrospective design with a moderate sample size, and incomplete representation of all viral variants.

Second, all patients were examined only once without dedicated follow up examinations.

Third, heterogeneous CT protocols across different scanners and the inclusion of both non-contrast and contrast-enhanced CT examinations may have increased imaging variability. To account for the potential influence of contrast administration, an additional sensitivity analysis restricted to non-contrast-enhanced CT examinations was performed. However, differences in other acquisition parameters and reconstruction protocols could not be fully controlled for.

Fourth, the viral subgroups were examined during different, largely non-overlapping periods of the pandemic, resulting in substantial confounding between viral variant and calendar time. Given the near-complete collinearity between variant and calendar time, statistical adjustment for examination month would not reliably separate these effects. Potential confounders, including vaccination status, smoking, and treatment, patient selection and disease stage at CT examination were not considered. In particular, the lack of information on prior immunological status limits the interpretation of the higher (less negative) Mean HU of Opacity values observed in the Delta subgroup, as a potential influence of vaccination or previous SARS-CoV-2 infection on these CT findings cannot be excluded. The persistence of the attenuation differences in the non-contrast-enhanced CT subgroup suggests that contrast administration alone does not explain the findings.

Our observed correlations and clustering patterns do not necessarily imply causation and time-dependent confounding cannot be excluded. Furthermore, as no multivariable prognostic analyses were performed, the observed correlations should be interpreted as associations and do not establish independent prognostic value. Therefore, observed differences between the subgroups cannot be attributed solely to variant-specific pathogenicity. Nevertheless, the observed differences may serve as a starting point for larger, independent studies that appropriately account for these potential confounders.

Fifth, the software requires human corrections in nearly all cases. The corrections were in some cases minimal, but in others, significant corrections were necessary. Excluding examinations requiring more than 20 min of correction (*n* = 3) may have underrepresented highly complex cases. As these examinations were not further evaluated after exclusion, their potential impact on the study results cannot be assessed retrospectively.

Although the analyses were reviewed by a second reader, no independent repeat quantification was performed. Therefore, interreader variability could not be assessed, and potential observer-dependent effects cannot be excluded. Here also the individual examiner’s experience should also be considered as a confounder and limitation.

Overall future multicenter studies with larger and more representative cohorts are needed. The retrospective design confines analysis to a specific period, excluding consideration of dynamic changes or disease stage.

## 5. Conclusions

The CT Pneumonia Analysis Prototype enables quantitative segmentation of CT imaging data across the COVID-19 subgroups wild-type, Alpha variant, and Delta variant and consistently identifies the corresponding examinations as COVID-19 pneumonia. However, the need for manual correction of the automated segmentations emphasizes that radiological oversight and curation remain essential. With such expert supervision, AI-assisted analysis facilitates the quantitative characterization of subgroup-specific differences. Given the retrospective observational design, these findings should be interpreted in light of potential confounding and heterogeneity in CT acquisition protocols.

Future studies combining semiquantitative CT, clinical and laboratory variables into AI-supported segmentation algorithms may further evaluate their potential relevance for disease severity and clinical outcome.

## Figures and Tables

**Table 1:** Detailed subgroup distribution of clinical parameters. ICU, Intensive Care Unit; NIV, non-invasive ventilation; HFOT, high-flow oxygen therapy.

Clinical Course	Wild-Type (*n* = 93)		Alpha (*n* = 66)		Beta/Gamma (*n* = 3)		Delta (*n* = 93)	
	*n*	%	N	%	*n*	%	*n*	%
ICU admission	36	38.7	25	37.9	2	66.7	29	31.2
Oxygen supplementation (nasal cannula, mask)	64	68.8	52	78.8	1	33.3	70	75.3
Non-invasive respiratory support (NIV, HFOT)	31	33.3	20	30.3	2	66.7	31	33.3
Invasive mechanical ventilation (intubation, tracheostomy)	13	14.0	11	16.7	1	33.3	12	12.9
Outcome—deceased	14	15.1	12	18.2	0	0	11	11.8
Outcome—recovered	69	74.2	44	66.7	1	33.3	67	72.0
Outcome—transferred	9	9.7	10	15.2	2	66.7	15	16.1
Outcome—missing data	1	1.1						

**Table 2:** Overview of analyzed laboratory parameters and statistically significant differences among subgroups. Data are presented as mean ± standard deviation (SD). The reported *p*-values are unadjusted results of Kruskal–Wallis tests. Nominal significance levels are indicated by * (*p* < 0.05) and ** (*p* < 0.01).

Laboratory Parameters	Wild-Type (*n* = 93)		Alpha (*n* = 66)		Delta (*n* = 93)		*p*
	(Mean ± SD)	*n*	(Mean ± SD)	*n*	(Mean ± SD)	*n*	
Creatine kinase (CK) (U/L)	333.43 ± 880.50	84	375.69 ± 698.08	59	389.62 ± 951.70	86	0.185
Troponin T (ng/L)	30.29 ± 44.51	90	27.89 ± 36.98	62	59.32 ± 224.34	87	0.710
Lactate dehydrogenase (LDH) (U/L)	405.03 ± 223.57	91	441.92 ± 211.65	65	402.36 ± 195.10	91	0.155
Aspartate aminotransferase (AST) (U/L)	54.31 ± 61.35	90	63.13 ± 38.17	61	58.69 ± 38.59	91	0.0099 **
Alanine aminotransferase (ALT) (U/L)	41.91 ± 53.50	88	42.80 ± 26.65	64	37.85 ± 27.79	92	0.089
C-reactive protein (CRP) (mg/L)	87.56 ± 82.61	91	93.87 ± 75.30	66	97.00 ± 74.07	92	0.334
Leukocyte count (Giga/L)	7.83 ± 5.21	89	6.89 ± 3.76	66	6.73 ± 2.88	91	0.722
Absolute lymphocyte count (Gigal/L)	0.98 ± 1.32	86	0.86 ± 0.45	66	0.89 ± 0.51	88	0.752
Relative lymphocyte count (%)	14.18 ± 10.81	86	14.84 ± 9.03	66	14.57 ± 8.38	88	0.536
D-dimer (mg/FEU)	1.71 ± 1.36	89	1.80 ± 4.09	63	1.89 ± 3.83	81	0.001 **
Procalcitonin (µg/L)	0.33 ± 0.54	80	0.23 ± 0.32	60	0.39 ± 0.64	74	0.298
Interleukin 6 (pg/mL)	121.45 ± 138.98	23	87.66 ± 108.20	41	239.07 ± 885.84	31	0.175
Venous pH	7.39 ± 0.09	82	7.42 ± 0.06	58	7.40 ± 0.06	78	0.026 *
Venous partial pressure of carbon dioxide (mmHg)	43.00 ± 12.43	82	39.41 ± 6.34	58	39.70 ± 6.39	77	0.069
Venous partial pressure of oxygen (mmHg)	43.07 ± 22.65	82	45.28 ± 18.48	58	47.99 ± 26.43	78	0.369
Oxygen saturation (%)	60.40 ± 22.81	72	68.37 ± 21.92	52	67.93 ± 23.52	67	0.089
Lactate (mmol/L)	2.34 ± 2.43	77	2.20 ± 1.39	53	1.87 ± 1.31	72	0.106

**Table 3:** Global and lobar-specific analysis of COVID-19 pneumonia in the wild-type, Alpha variant, and Delta variant using COVID-19 probability, Mean HU of Opacity, Volume of Opacity and Percentage of Opacity. Data are presented as mean ± standard deviation (SD). For global mean opacity attenuation (both lungs), valid measurements were available for 92 wild-type, 66 Alpha, and 89 Delta patients. The reported *p*-values are unadjusted results of Kruskal–Wallis tests. Nominal significance levels are indicated by * (*p* < 0.05) and ** (*p* < 0.01). COVID-19, coronavirus disease 2019.

	Wild-Type	Alpha	Delta	*p*
COVID-19 Probability	0.80 ± 0.36	0.85 ± 0.32	0.88 ± 0.30	0.059
Mean HU of Opacity (Mean ± SD)				
Both Lungs (BL)	−475.12 ± 116.47	−464.68 ± 114.80	−423.92 ± 112.56	0.002 **
Left Lung (LL)	−456.01 ± 150.97	−471.71 ± 133.18	−413.39 ± 130.31	0.004 **
Right Lung (RL)	−464.41 ± 142.32	−462.08 ± 117.67	−404.87 ± 143.85	0.002 **
Left Upper Lobe (LUL)	−453.78 ± 202.20	−493.98 ± 163.71	−415.04 ± 182.23	0.009 **
Left Lower Lobe (LLL)	−408.31 ± 177.33	−444.65 ± 131.64	−383.10 ± 140.51	0.021 *
Right Upper Lobe (RUL)	−468.37 ± 184.98	−453.26 ± 175.39	−406.63 ± 177.29	0.007 **
Right Middle Lobe (RML)	−486.60 ± 225.52	−530.36 ± 141.72	−428.63 ± 205.42	0.006 **
Right Lower Lobe (RLL)	−415.43 ± 164.46	−427.55 ± 136.38	−375.74 ± 151.72	0.045 *
Volume of Opacity (Mean ± SD)				
Both Lungs (BL)	984.64 ± 908.92	1161.17 ± 933.55	988.19 ± 866.20	0.339
Left Lung (LL)	426.12 ± 423.58	485.24 ± 423.16	442.90 ± 429.22	0.424
Right Lung (RL)	558.52 ± 510.51	675.93 ± 541.16	545.29 ± 468.67	0.239
Left Upper Lobe (LUL)	198.76 ± 223.74	218.62 ± 232.76	190.33 ± 224.37	0.491
Left Lower Lobe (LLL)	229.51 ± 217.55	264.28 ± 205.99	252.57 ± 222.87	0.334
Right Upper Lobe (RUL)	209.38 ± 209.65	243.52 ± 217.55	186.46 ± 180.18	0.322
Right Middle Lobe (RML)	74.55 ± 90.73	86.45 ± 87.69	69.94 ± 77.12	0.258
Right Lower Lobe (RLL)	289.82 ± 248.12	345.96 ± 217.90	288.83 ± 248.50	0.309
Percentage of Opacity (Mean ± SD)				
Both Lungs (BL)	27.84 ± 26.54	33.44 ± 26.08	30.02 ± 25.27	0.257
Left Lung (LL)	26.45 ± 26.85	30.81 ± 25.68	29.37 ± 26.17	0.286
Right Lung (RL)	29.43 ± 27.51	35.90 ± 28.11	31.15 ± 26.46	0.229
Left Upper Lobe (LUL)	23.15 ± 26.70	25.93 ± 26.41	23.22 ± 25.33	0.471
Left Lower Lobe (LLL)	31.67 ± 30.04	38.06 ± 27.42	37.64 ± 29.79	0.181
Right Upper Lobe (RUL)	27.68 ± 28.61	34.17 ± 31.49	27.74 ± 26.33	0.362
Right Middle Lobe (RML)	23.36 ± 27.69	27.82 ± 26.41	23.21 ± 25.17	0.274
Right Lower Lobe (RLL)	36.87 ± 31.65	42.96 ± 30.06	38.82 ± 30.91	0.376

**Table 4:** Exploratory pairwise comparisons of mean opacity attenuation across SARS-CoV-2 variant groups by lung region. Unadjusted two-sided *p*-values from Mann–Whitney U tests are reported. Nominal significance levels are indicated by * (*p* < 0.05) and ** (*p* < 0.01). No correction for multiple comparisons was applied to the *p*-values presented in this table. The global (both lungs) comparison was updated after exclusion of five technically non-evaluable attenuation measurements. WT, wild-type; SARS-CoV-2, Severe Acute Respiratory Syndrome Coronavirus 2.

Lung Region	WT vs. Alpha *p*	WT vs. Delta *p*	Alpha vs. Delta *p*
Both Lungs (BL)	0.419	<0.001 **	0.026 *
Left Lung (LL)	0.823	0.003 **	0.007 **
Right Lung (RL)	0.495	0.001 **	0.015 *
Left Upper Lobe (LUL)	0.505	0.019 *	0.005 **
Left Lower Lobe (LLL)	0.598	0.039 *	0.009 **
Right Upper Lobe (RUL)	0.407	0.002 **	0.041 *
Right Middle Lobe (RML)	0.083	0.006 **	0.006 **
Right Lower Lobe (RLL)	0.960	0.060	0.043 *

**Table 5:** Subgroup-specific correlations between semiquantitative lung parameters and clinical parameters. Spearman’s correlation coefficients (r) and unadjusted *p*-values are reported. Nominal significance levels are indicated by * (*p* < 0.05) and ** (*p* < 0.01). No correction for multiple comparisons was applied.

Correlation of Quantification Parameters and Clinical Parameters	Wild-Type		Alpha		Delta	
	r	*p*	r	*p*	r	*p*
Volume of Opacity						
Length of Hospital stay	0.376	<0.001 **	0.297	0.016 *	0.219	0.039 *
Length of ICU stay	0.672	0.001 **	0.414	0.040 *	0.159	0.409
Duration of invasive ventilation	0.241	0.428	0.288	0.419	0.007	0.983

**Table 6:** Subgroup-specific correlations between semiquantitative lung parameters and laboratory measures. Spearman’s correlation coefficients (r) and unadjusted *p*-values are reported. Nominal significance levels are indicated by * (*p* < 0.05) and ** (*p* < 0.01). No correction for multiple comparisons was applied.

Correlation of Quantification Parameters and Laboratory Parameters	Wild-Type		Alpha		Delta	
	r	*p*	r	*p*	r	*p*
Volume of Opacity						
C-reactive protein	0.681	<0.001 **	0.634	<0.001 **	0.536	<0.001 **
Leukocyte count	0.216	0.042 *	0.398	0.001 **	0.389	<0.001 **
Relative lymphocyte count	−0.326	0.002 **	−0.549	<0.001 **	−0.225	0.035 *
Aspartate aminotransferase	0.492	<0.001 **	0.517	<0.001 **	0.578	<0.001 **
D-dimer	0.105	0.330	0.143	0.264	0.088	0.434

## Data Availability

The strict requirements at our university hospital regarding data policy and data protection do not allow data sharing.

## Supplementary Materials

The following supporting information can be downloaded at https://www.mdpi.com/article/10.3390/diagnostics16213696/s1: Table S1: CT acquisition characteristics by SARS-CoV-2 variant.

## Abbreviations

The following abbreviations are used in this manuscript:

AI	Artificial intelligence
ALT	Alanine aminotransferase
AST	Aspartate aminotransferase
BL	Both Lung
CAD	Computer-aided detection
CK	Creatine kinase
CNN	Convolutional neural network
COPD	Chronic obstructive pulmonary disease
COVID-19	Coronavirus disease 2019
CRP	C-reactive protein
CT	Computed tomography
DICOM	Digital Imaging and Communications in Medicine
HFOT	High-flow oxygen therapy
HU	Hounsfield Units
ICU	Intensive care unit
IL-6	Interleukin-6
LDH	Lactate dehydrogenase
LLL	Left Lower Lobe
LUL	Left Upper Lobe
N	Number of patients
NIV	Non-invasive ventilation
pCO_2_	Partial pressure of carbon dioxide
PCR	Polymerase chain reaction
PGMI	Perfect, Good, Moderate, Inadequate
pO_2_	Partial pressure of oxygen
RLL	Right Lower Lobe
RML	Right Middle Lobe
RT-PCR	Reverse transcription polymerase chain reaction
RUL	Right Upper Lobe
SAP	Systems, Applications and Products
SARS-CoV-2	Severe Acute Respiratory Syndrome Coronavirus 2
SD	Standard deviation
WHO	World Health Organization
WT	Wild-type
