# Supplementary material for: AI-Supported Semiquantitative CT Characterization of COVID-19 Pneumonia Across SARS-CoV-2 Wild-Type, Alpha, and Delta Variants and Its Association with Clinical Parameters
Source: Diagnostics (Basel). 2026 Nov 9;16(21):3696. doi: 10.3390/diagnostics16213696 (PMC13762066; doi:10.3390/diagnostics16213696)
Supplement: Supplementary file 1 [file diagnostics-16-03696-s001.zip › diagnostics-4602097-supplementary.pdf]

Table S1: CT acquisition characteristics by SARS-CoV-2 variant

CT image acquisition	Wild-type		Alpha		Delta	
	n	%	n	%	n	%
**CT scanner models**						
Siemens EDGE Plus	77	82.8	54	81.8	77	82.2
Siemens AS+	3	3.2	1	1.5	6	6.5
Siemens Force	6	6.5	8	12.1	9	9.7
Toshiba Aquilion	6	6.5			1	1.1
Siemens Biograph 40	1	1.1	1	1.5		
Siemens go.TOP			1	1.5		
Siemens Edge			1	1.5		
**Slice thickness (mm)**						
0,75	25	26.9	28	24.4	6	6.5
1.0	60	64.5	37	56.1	87	93.5
2.0	1	1.1				
3.0	2	2.2	1	1.5		
5.0	5	5.4				
**Contrast agent**						
no	69	74.2	36	54.5	55	59.1
yes	24	25.8	30	45.5	38	40.9
